# Down-Regulation of Protein Kinase Cδ Inhibits Inducible Nitric Oxide Synthase Expression through IRF1

**DOI:** 10.1371/journal.pone.0052741

**Published:** 2013-01-09

**Authors:** Tiina Leppänen, Riku Korhonen, Mirka Laavola, Riina Nieminen, Raimo K. Tuominen, Eeva Moilanen

**Affiliations:** 1 The Immunopharmacology Research Group, University of Tampere School of Medicine and Tampere University Hospital, Tampere, Finland; 2 The Division of Pharmacology and Toxicology, University of Helsinki Faculty of Pharmacy, Helsinki, Finland; University of Delhi, India

## Abstract

In inflammation, pro-inflammatory cytokines and bacterial products induce the production of high amounts of NO by inducible nitric oxide synthase (iNOS) in inflammatory and tissue cells. NO is an effector molecule in innate immunity, and it also has regulatory and pro-inflammatory/destructive effects in the inflammatory process. Protein kinase Cδ (PKCδ) is an important signaling protein regulating B lymphocyte functions, but less is known about its effects in innate immunity and inflammatory gene expression. In the present study we investigated the role of PKCδ in the regulation of iNOS expression in inflammatory conditions. NO production and iNOS expression were induced by LPS or a combination of cytokines IFNγ, IL-1β, and TNFα. Down-regulation of PKCδ by siRNA and inhibition of PKCδ by rottlerin suppressed NO production and iNOS expression in activated macrophages and fibroblasts. PKCδ directed siRNA and inhibition of PKCδ by rottlerin suppressed also the expression of transcription factor IRF1, possibly through inhibition of STAT1 activation. Accordingly, down-regulation of IRF1 by siRNA reduced iNOS expression in response to inflammatory stimuli. In addition, inhibition of PKCδ showed anti-inflammatory effects in carrageenan induced paw inflammation in mice as did iNOS inhibitor L-NIL. These results suggest that inhibitors of PKCδ have anti-inflammatory effects in disease states complicated by enhanced NO production through iNOS pathway.

## Introduction

Nitric oxide (NO) is a gaseous signaling molecule that regulates various physiological and pathophysiological processes in the human body. The production of NO is increased in inflammation, and it is known to act as a regulatory and pro-inflammatory modulator in several inflammatory diseases [1]–[4]. NO is synthesized from l-arginine by three nitric oxide synthase (NOS) enzymes; endothelial NOS (eNOS), inducible NOS (iNOS), and neuronal NOS (nNOS). eNOS and nNOS are constitutively expressed and responsible for the low physiological production of NO. Expression of iNOS is induced in response to e.g. bacterial products and pro-inflammatory cytokines. Once expressed, iNOS produces high amounts of NO for a prolonged period of time [2], [5]. iNOS expression is regulated mainly at transcriptional level, but also post-transcriptional regulation has been reported [1], [3], [6]. Nuclear factor κB (NF-κB), and interferon response factor-1 (IRF1) are important transcription factors in iNOS expression [7]–[11]. Compounds that inhibit iNOS activity or iNOS expression have anti-inflammatory properties in various *in vitro* and *in vivo* models [12].

Protein kinase C (PKC) is a family of serine-threonine protein kinase isoenzymes that represent one of the major signal transduction systems in inflammation. Based on the differences in the chemical structure and substrate requirements, the PKC isoenzymes have been classified into three groups. Conventional isoenzymes (α, βI, βII, and γ) are calcium dependent, and they require diacylglycerol and phosphatidylserine for activation. Novel isoenzymes (δ, ε, η and θ) are calcium independent, but need diacylglycerol and phosphatidylserine for activation. Atypical isoenzymes (ξ and 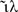) are independent of both calcium and diacylglycerol for activation [13], [14].

The most extensively studied isoenzyme of the novel group is PKCδ. It is ubiquitously expressed and has been shown to regulate cell growth, differentiation and apoptosis, and immune response [15], [16]. Studies with PKCδ knockout mice show that PKCδ is an important regulator of B lymphocyte functions [17], [18]. PKCδ knockout mice develop and reproduce normally but have increased number of B cells in spleen and other peripheral organs. The mice die prematurely due to a severe autoimmune disease, and the alterations in B cells suggest that PKCδ plays a role in the production of immunological tolerance [19]. Less is known about the role of PKCs, especially PKCδ in innate immunity and in the regulation of expression of inflammatory genes in activated macrophages and tissue cells. The aim of the present study was to investigate the hypothesis that PKCδ is involved in the regulation of iNOS expression in inflammatory conditions. The results suggest that PKCδ up-regulates the expression of transcription factor IRF1, possibly through activation of transcription factor STAT1 (signal transducer and activator of transcription 1). This is further reflected as enhanced expression of iNOS in activated macrophages and fibroblasts. The role of PKCδ in the development of acute inflammation also *in vivo* was supported by the present finding that PKCδ inhibitor rottlerin suppressed carrageenan induced paw inflammation in the mouse, as did iNOS inhibitor L-NIL.

## Materials and Methods

### Materials

Reagents were purchased as follows: rabbit polyclonal iNOS, β-actin, lamin A/C, and PKCδ antibodies and HPR-conjugated goat polyclonal anti-rabbit IgG antibodies were from Santa Cruz Biotechnology Inc. (Santa Cruz, CA, USA). IRF1 antibody was from R&D Systems Europe Ltd (Abingdon, UK). PKCδ siRNA, IRF1 siRNA, non-targeting control siRNA, and DharmaFECT transfection reagents were from Thermo Fisher Scientific (Lafayette, CO, USA). Rottlerin and all other reagents were from Sigma-Aldrich (St. Louis, MO, USA).

### Cell culture

Murine J774.2 macrophages (European Collection of Cell Cultures, Porton Down, Wiltshire, UK) were cultured at 37°C in 5% CO_2_ atmosphere and grown in Dulbecco's modified Eagle's medium with Ultraglutamine 1 (Lonza, Verviers Sprl, Verviers, Belgium) supplemented with 5% heat-inactivated foetal bovine serum (Lonza), 100 U/ml penicillin, 100 µg/ml streptomycin and 250 ng/ml amphotericin B (all from Invitrogen, Paisley, UK).

Murine L929 fibroblasts (CCL-1; American Type Culture Collection, Manassas, VA, USA) were cultured at 37°C in 5% CO_2_ atmosphere and grown in Eagle's minimum essential medium with l-glutamine containing 10% heat-inactivated foetal bovine serum and supplemented with sodium bicarbonate (0.15%), non-essential amino acids (1 mM each), sodium puryvate (1 mM) (all from Lonza) and 100 U/ml penicillin, 100 µg/ml streptomycin and 250 ng/ml amphotericin B (all from Invitrogen).

Cells were seeded on 24-well plates for siRNA and Western blot experiments, RT-PCR, ELISA, and nitrite measurements, and on 10 cm dishes for extraction of nuclear proteins. Cells were grown for 48 h (L929) or 72 h (J774) to confluence prior to the experiments.

Cytotoxicity of tested compounds was ruled out by measuring cell viability using Cell Proliferation Kit II (Roche Diagnostics, Mannheim, Germany).

### NF-κB, GAS (STAT1) and IRF1 reporter experiments

The luciferase reporter constructs to study NF-κB [pNFκB(luc)neo] and STAT1 [pGAS(luc)neo] mediated transcription were provided by Professor Hartmut Kleinert at the Johannes Gutenberg University, Mainz, Germany. pNFκB(luc)neo contained five NF-κB binding sites and pGAS(luc)neo four STAT1 binding γ-activated sites (GASs) to drive luciferase expression. The plasmids contained a neomycin resistance gene under the control of TK promoter for mammalian selection.

For NF-κB reporter experiments, L929 cells were stably transfected with pNFκB(luc)neo reporter plasmid using Lipofectamine 2000 (Invitrogen) according to the manufacturer's instructions. Transfected cells were selected with G 418 disulphate salt (800 µg/ml) treatment (Sigma–Aldrich). After the selection, the survived clones were pooled to give rise to L929 pNF-κB cell line and further cultured in the presence of 400 µg/ml of G 418.

For STAT1 reporter experiments, L929 cells were transiently transfected with pGAS(luc)neo reporter plasmid using Lipofectamine 2000 according to the manufacturer's instructions. Briefly, transfection complexes were prepared by mixing 0.8 µg of plasmid DNA and 2 µL of Lipofectamine 2000 in 100 µL of serum-free culture medium without antibiotics, and the mix was incubated in room temperature for 30 min. The mix containing transfection complexes was then added to the cells, and the cells were further incubated for 24 h before the experiments were started. After the experiments, luciferase activity was measured by luminometer using Luciferase Assay System (Promega, Madison, WI, USA).

IRF1 reporter experiments were carried out by using IRF1 Cignal™ Pathway Reporter Kit (QIAGEN, Helsinki, Finland). L929 cells were transfected with IRF1 reporter according to the manufacturer's instructions. Transfection complexes were prepared by mixing 0.2 µg of plasmid DNA and 0.5 µL of Lipofectamine 2000 in 50 µL of serum-free culture medium without antibiotics, and the mix was incubated in room temperature for 30 min. Transfection complexes were then added to the cells, and the cells were further incubated for 24 h before the experiments were started. After the experiments, firefly and *Renilla* luciferase activities were measured by luminometer using Dual-Glo®Luciferase Assay System (Promega).

### Down-regulation of PKCδ and IRF1 by siRNA

PKCδ expression in L929 cells was down-regulated using Dharmacon ON TARGET plus SMARTpool siRNA oligos. Cells were grown to ∼80% confluence and transfected with PKCδ-specific siRNA or non-targeting control siRNA using DharmaFECT 1 transfection reagent according to manufacturer's instructions (Thermo Fisher Scientific).

In J774 cells the expression of PKCδ and IRF1 were down-regulated using Dharmacon ON TARGET plus siRNA oligos (J-040147-06 and L-046743-01, respectively). Cells were grown to ∼80% confluence and transfected with PKCδ or IRF1siRNA or non-targeting control siRNA using DharmaFECT 4 transfection reagent according to manufacturer's instructions (Thermo Fisher Scientific). After 24 h incubation, the transfection medium was replaced with fresh culture medium.

Forty-eight hours after the transfection, the experiments were started and stimulants with or without rottlerin were added in fresh culture medium. Down-regulation of the target geneby siRNA was determined from samples extracted at the beginning of the experiments. Down-regulation of PKCδ and IRF1 by siRNA was approximately 80% as compared to those with non-targeting control siRNA (Figure S1).

### Nitrite assays

The effects of the tested compounds on the ability of the cells to produce NO was determined by measuring the accumulation of nitrite, a stable metabolite of NO, in the culture medium by the method of Griess [20].

### Western blotting

At indicated time points, cells were rapidly washed with ice-cold phosphate-buffered saline (PBS) and solubilized in cold lysis buffer containing 10 mM Tris-base, pH 7.4, 5 mM EDTA, 50 mM NaCl, 1% Triton X-100, 0.5 mM phenylmethylsulfonyl fluoride, 1 mM sodiumorthovanadate, 20 µg/ml leupeptin, 50 µg/ml aprotinin, 5 mM NaF, 2 mM sodiumpyrophosphate and 10 µM n-octyl-β-D-glucopyranoside. After incubation for 15 min on ice, cell lysates were centrifuged (13 400× *g*, 4°C, 10 min), supernatants were collected and stored in SDS sample buffer in −20°C. An aliquot of the supernatant was used to determine protein concentration by the Coomassie blue method [21].

Protein samples (20 µg of lysates) were analyzed according to standard Western blotting protocol as described previously [22]. The membrane was incubated with the primary antibody in the blocking solution at 4°C overnight, and with the secondary antibody in the blocking solution for 1 h at room temperature. Bound antibody was detected using Super Signal® West Pico or Dura chemiluminescent substrate (Pierce, Rockford, USA) and Image Quant LAS 4000 mini imaging system (GE Healthcare Bio-Sciences AB). The quantitation of the chemiluminescent signal was carried out with the use of Image Quant TL software (GE Healthcare).

### Electrophoretic mobility shift assay (EMSA)

Nuclear extracts for NF-κB EMSA were extracted as described previously [23]. Cells were incubated with lipopolysaccharide (LPS, 10 ng/ml) in the absence and presence of rottlerin (10 µM) for 1 h prior to the extraction of nuclear proteins.

Transcription factor consensus oligonucleotides for NF-κB (Promega, Madison, WI, USA) were 5′-^32^P-end-labeled with DNA 5′-End Labeling Kit (Roche Diagnostics, Indianapolis, IN, USA). For binding reactions, 5 µg of nuclear extract was incubated in 20 µl of total reaction volume containing 0.1 mg/ml (poly)dI-dC, 1 mM dithiothreitol, 10 mM Tris-HCl, pH 7.5, 1 mM EDTA, 40 mM KCl, and 10% glycerol for 20 min in room temperature. ^32^P-labeled oligonucleotide probe (0.2 ng) was added and the reaction mixture was incubated for 10 min. Protein - DNA complexes were separated from DNA probe by electrophoresis on a native 4% polyacrylamide gel. The gel was dried and autoradiographed using intensifying screen at −70°C.

### RNA extraction and quantitative RT-PCR

Total RNA extraction was carried out with the use of GenElute™ Mammalian Total RNA Miniprep Kit (Sigma-Aldrich). Reverse-transcription of RNA to cDNA and PCR reactions were performed as previously described [23]. For luciferase mRNA experiments, total RNA was treated with DNase I (Fermentas UAB, Vilnius, Lithuania) prior to conversion to cDNA.

Primers and probes (Table S1) for luciferase, iNOS, interleukin-6 (IL-6), tumor necrosis factor α (TNFα), and glyceraldehyde-3-phosphate dehydrogenase (GAPDH, used as a control gene) were designed using Primer Express® Software (Applied Biosystems, Foster City, CA, USA) and supplied by Metabion (Martinsried, Germany). Expression of IRF1 mRNA was measured using TaqMan® Gene Expression Assay (Applied Biosystems, Foster City, CA, USA).

### Actinomycin D assay

Actinomycin D assay was performed to study the decay of iNOS mRNA. L929 fibroblasts were incubated with a mixture of cytokines interleukin-1β (IL-1β), interferon γ (IFNγ), and TNFα (each 10 ng/ml) in the absence and presence of rottlerin (3 µM) for 6 h before the addition of actinomycin D (2 µg/ml), an inhibitor of transcription. Thereafter, RNA was extracted at indicated time points and subjected to quantitative RT-PCR to measure the remaining mRNA.

### IL-6 and TNFα ELISA

IL-6 and TNFα were measured in the culture medium by enzyme linked immunosorbent assay (ELISA) using reagents from R&D Systems Europe Ltd (Abingdon, UK).

### Carrageenan induced paw edema in mice

Anti-inflammatory effects of rottlerin *in vivo* were studied by measuring carrageenan induced paw edema in male C57BL/6 mice (bred at the University of Tampere). The study was carried out in accordance with the legislation for the protection of animals used for scientific purposes (directive 2010/63/EU) and approved by the National Animal Experiment Board (approval number ESLH-2009-07700/Ym-23, granted September 23, 2009). Paw edema was induced under anesthesia and all efforts were made to minimize suffering.

Animals were housed under conditions of optimum light, temperature and humidity (12∶12 h light–dark cycle, 22±1°C, 50–60%) with food and water provided ad libitum. Mice were randomly divided into three groups: control group, L-NIL group (50 mg/kg) and rottlerin group (10 mg/kg), with 6 mice in each group. Two hours before carrageenan the mice were treated with 200 µl of normal saline or the drug tested by intraperitoneal injection. The mice were anesthesized by intraperitoneal injection of 0.5 mg/kg of medetomide (Domitor® 1 mg/ml. Orion Oyj, Espoo, Finland) and 75 mg/kg of ketamine (Ketalar® 10 mg/ml, Pfizer Oy Animal Health, Helsinki, Finland), and thereafter the mice received a 30 µl intradermal injection in one hindpaw of normal saline containing λ-carrageenan (1.5%). The contralateral paw received 30 µl of saline and it was used as a control. Edema was measured before and three and six hours after carrageenan injection by using plethysmometer (Ugo Basile, Comerio, Italy). Edema is expressed as the difference, in µl, between the volume changes of the carrageenan treated paw and the control paw.

### Statistics

Results are expressed as mean+standard error of mean (SEM). Statistical significance of the results was calculated by one-way ANOVA with Dunnett's or Bonferroni's post test. Differences were considered significant at *p<0.05, **p<0.01, ***p<0.001.

## Results

### Effects of PKCδ on nitric oxide production and iNOS expression

**Figure 1:**
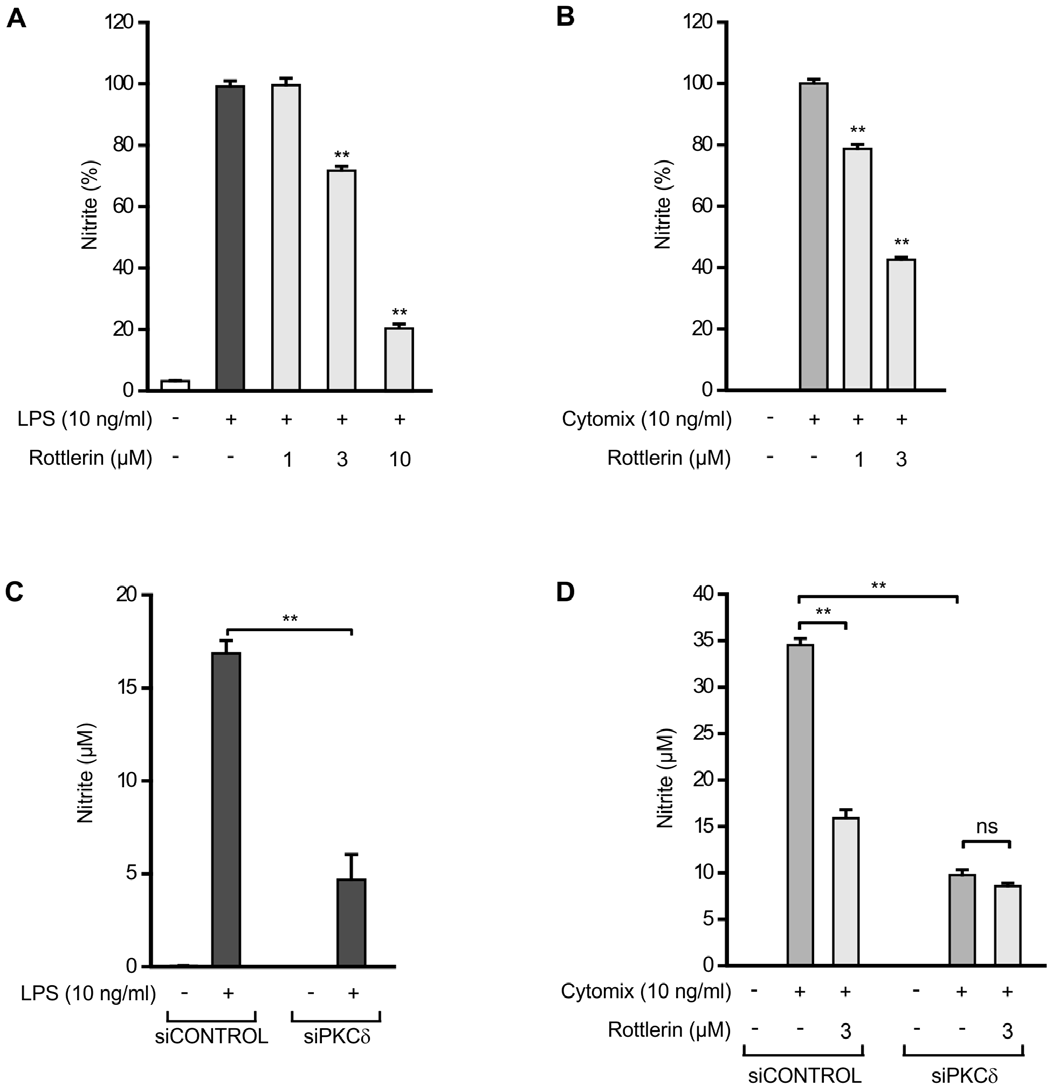
Effect of PKCδ on NO production. (A) Effects of rottlerin on LPS-induced NO production in J774 macrophages was measured after 24 h incubation as nitrite accumulated in the culture medium by the method of Griess. Values are mean+SEM, n = 4. (B) Effects of rottlerin on cytokine-induced (IL-1β, IFNγ, and TNFα) NO production in L929 fibroblasts after 24 h incubation. Values are mean+SEM, n = 4. (C) J774 macrophages were transiently transfected with PKCδ siRNA using DharmaFECT 4 transfection reagent. Treatment with non-targeting siRNA was used as control. Macrophages were stimulated with LPS for 24 h before the NO production was measured. Values are mean+SEM, n = 3. (D) L929 fibroblasts were transiently transfected with PKCδ siRNA using DharmaFECT 1 transfection reagent and treatment with non-targeting siRNA was used as control. L929 fibroblasts were stimulated with a combination of cytokines (IL-1β, IFNγ, and TNFα) and treated with PKCδ inhibitor rottlerin for 24 h before NO production was measured. Values are mean+SEM, n = 7,** p<0.01.

**Figure 2:**
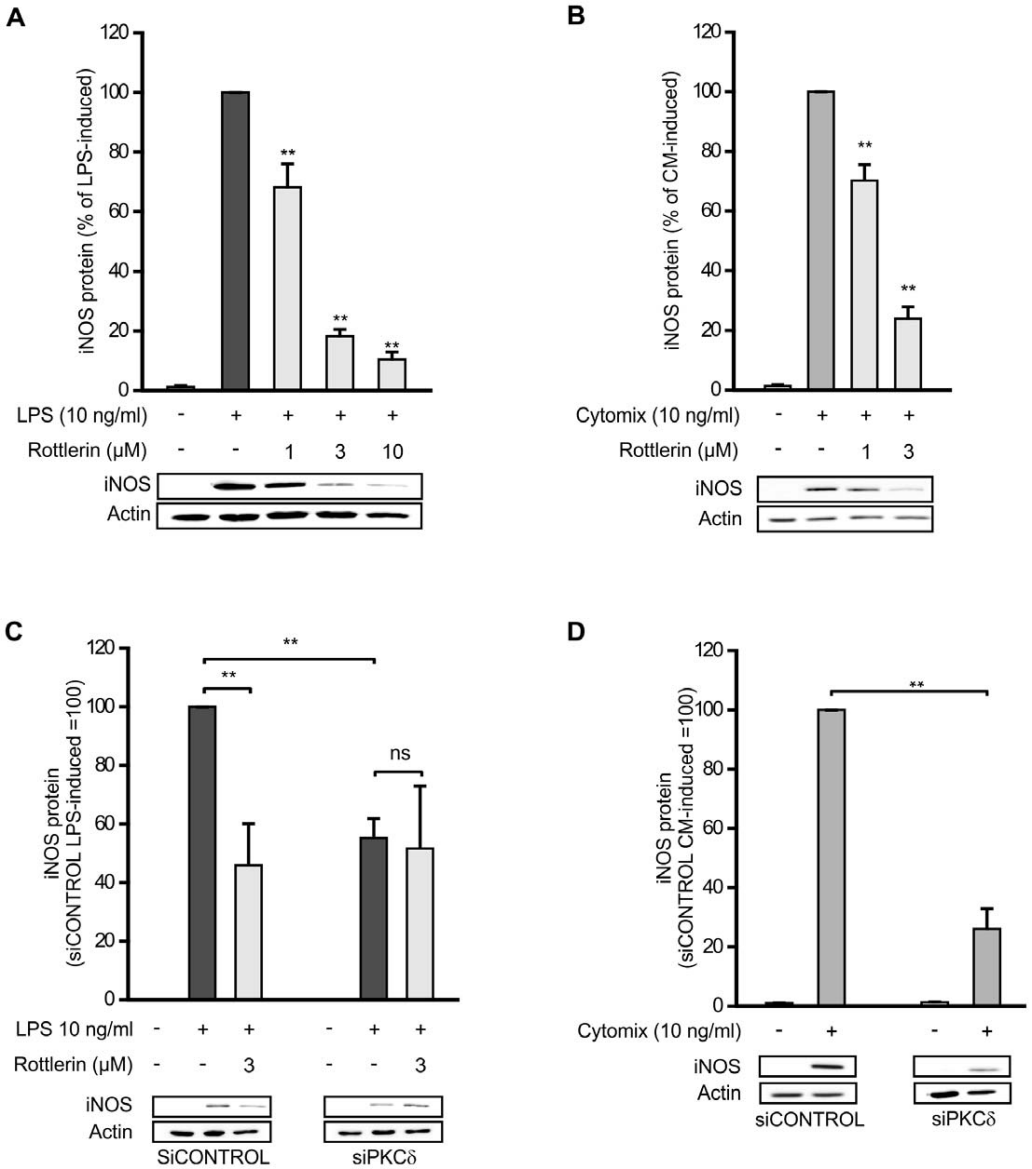
Effects of PKCδ on iNOS protein expression. (A) J774 macrophages were stimulated with LPS and treated with increasing concentrations of rottlerin. (B) L929 fibroblasts were stimulated with a combination of cytokines (IL-1β, IFNγ, and TNFα) and treated with increasing concentrations of rottlerin. (C) J774 macrophages were transiently transfected with PKCδ siRNA using DharmaFECT 4 transfection reagent and treatment with non-targeting siRNA was used as control. Thereafter the macrophages were stimulated with LPS and treated with rottlerin. (D) L929 fibroblasts were transiently transfected with PKCδ siRNA using DharmaFECT 1 transfection reagent and treatment with non-targeting siRNA was used as control. Thereafter L929 fibroblasts were stimulated with a combination of cytokines (IL-1β, IFNγ, and TNFα). After 24 h, incubations were terminated and immunoblots were run using iNOS specific antibody. Actin was determined as a loading control. Chemiluminescent signal was quantified as described under the Methods section. Values are mean+SEM, n = 3 (n = 4 in A), **p<0.01.

NO production and iNOS protein expression was induced by incubation with bacterial endotoxin LPS (10 ng/ml, J774 macrophages) or with a mixture of cytokines (IL-1β, IFNγ, and TNFα, 10 ng/ml each, L929 fibroblasts). To determine the effect of PKCδ inhibition on NO production and iNOS expression, the cells were treated with LPS or the mixture of cytokines in the absence and presence of rottlerin, an inhibitor of PKCδ for 24 h. Rottlerin decreased NO production (Fig. 1A–B) and iNOS expression (Fig. 2A–B) in a dose-dependent manner.

The effect of PKCδ on NO production and iNOS expression was also studied by silencing PKCδ with siRNA. PKCδ siRNA resulted in >80% suppression in PKCδ protein levels (Figure S1). Silencing of PKCδ clearly decreased NO production (Fig. 1C–D) and iNOS expression (Fig. 2C–D) in response to inflammatory stimuli as compared to cells treated with non-targeting control siRNA. In addition, when PKCδ was down-regulated with siRNA, rottlerin had no effect on NO production or iNOS expression suggesting that the inhibitory action of rottlerin on iNOS expression and NO production was most likely dependent on its inhibitory effect on PKCδ.

### Effects of PKCδ on the degradation of iNOS protein

**Figure 3:**
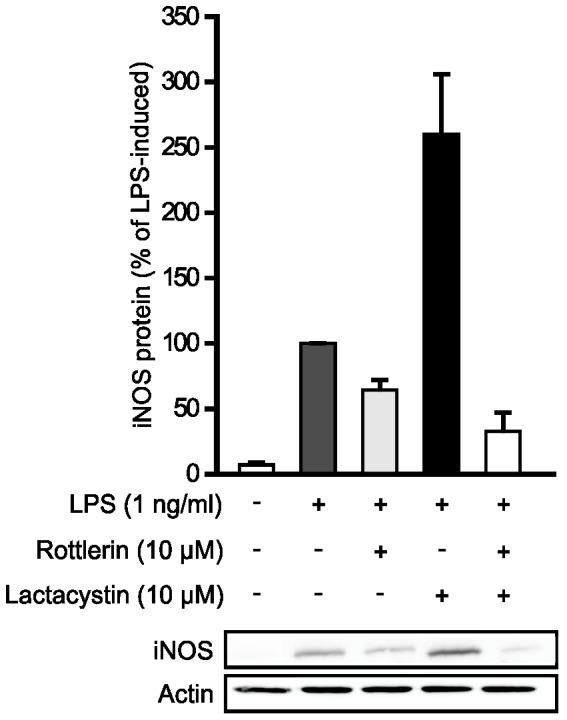
Effects of PKCδ inhibitor rottlerin on the degradation of iNOS protein. J774 macrophages were treated with LPS and rottlerin for 8 h before the addition of proteasome inhibitor lactacystin. After 6 h, incubations were terminated and immunoblots were run using iNOS specific antibody. Actin was determined as a loading control. Chemiluminescent signal was quantified as described under the Methods section. Values are mean+SEM, n = 3.

In order to find out whether PKCδ inhibition affects iNOS synthesis or degradation of iNOS protein, the effect of PKCδ inhibitor rottlerin on iNOS protein degradation was studied. Since iNOS protein has been shown to be degraded by the proteasome [24]–[26], we investigated the effect of rottlerin on LPS-induced iNOS expression in the presence of a proteasome inhibitor lactacystin. LPS increased the expression of iNOS protein and this was further enhanced by lactacystin (Fig. 3). However, rottlerin decreased the expression of iNOS protein in LPS treated cells as well as in LPS and lactacystin treated cells suggesting that rottlerin does not alter proteasome-mediated degradation of iNOS protein.

### Effects of PKCδ on iNOS mRNA expression and stability

**Figure 4:**
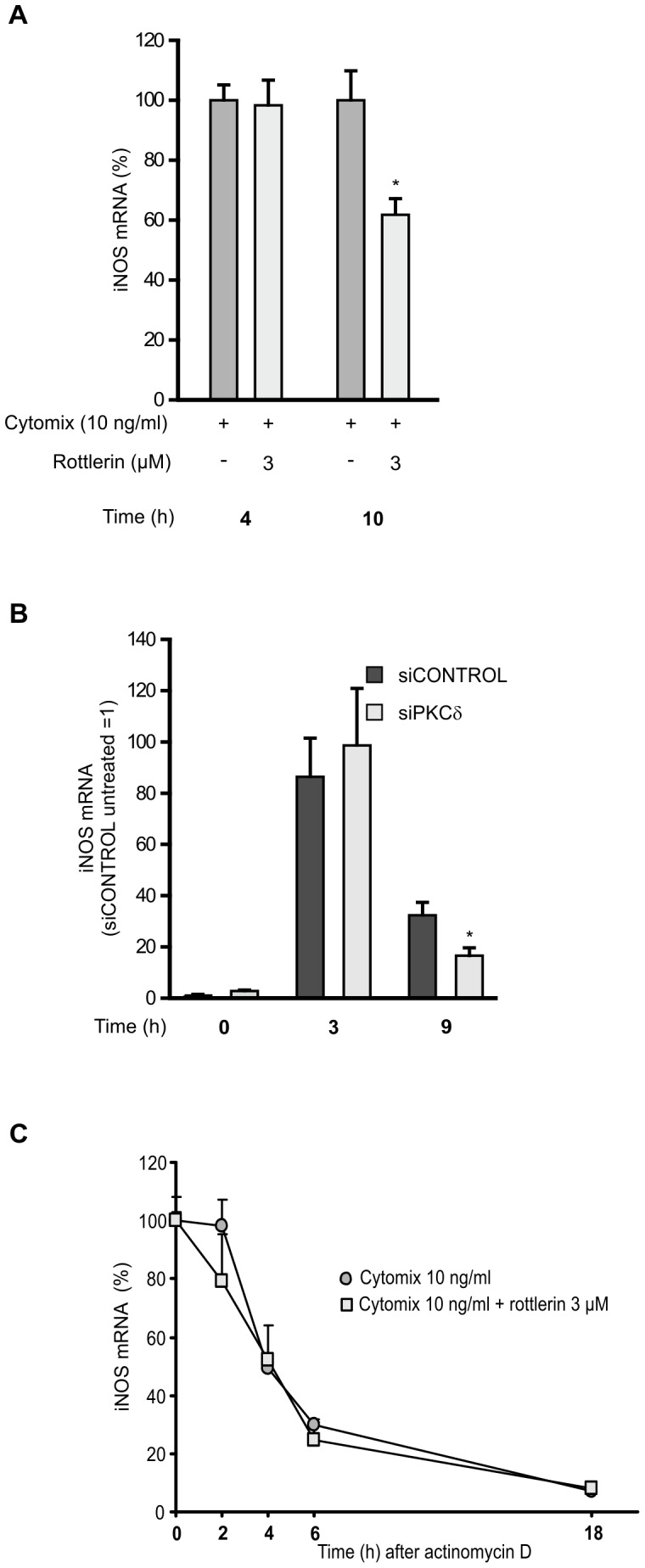
Effect of PKCδ on iNOS mRNA expression and stability. (A) L929 fibroblasts were stimulated with a combination of cytokines (IL-1β, IFNγ, and TNFα) and treated with PKCδ inhibitor rottlerin for 4 or 10 h. At indicated time points incubations were terminated and extracted total RNA was subjected to RT-PCR. (B) J774 macrophages were transiently transfected with PKCδ siRNA using DharmaFECT 4 transfection reagent. Treatment with non-targeting siRNA was used as control. Macrophages were stimulated with LPS for 3 or 9 h before incubations were terminated and extracted total RNA was subjected to RT-PCR. (C) L929 fibroblasts were activated by a combination of cytokines (IL-1β, IFNγ, and TNFα) and treated with rottlerin. After 6 h, actinomycin D (2 µg/ml) was added into the cell culture to stop transcription. Incubations were terminated at indicated time points after actinomycin D and extracted total RNA was subjected to RT-PCR. iNOS mRNA levels were normalized against GAPDH mRNA. Values are mean+SEM, n = 3. *p<0.05.

In the further studies, we investigated the effect of PKCδ inhibition on cytokine-induced expression of iNOS mRNA. Rottlerin had no effect on iNOS mRNA expression when measured at an early time point (4 h), but when measured at a later time point (10 h), rottlerin decreased iNOS mRNA expression significantly (Fig. 4A). Next we studied the effect of PKCδ down-regulation by siRNA on LPS-induced iNOS mRNA expression and in these experiments, we noticed a similar pattern. At an early time point PKCδ siRNA did not affect the level of iNOS mRNA, but at a later time point PKCδ siRNA decreased the level of iNOS mRNA when compared to cells transfected with non-targeting control siRNA (Fig. 4B).

In addition to the transcriptional regulation, iNOS expression is regulated also at the level of mRNA stability [27]–[30]. Therefore, the effect of PKCδ inhibition on the degradation of iNOS mRNA was studied in an actinomycin D assay. Cells were treated with the mixture of cytokines in the absence and presence of rottlerin (3 µM) for 12 h before the addition of actinomycin D (2 µg/ml), an inhibitor of transcription. Thereafter the cells were further incubated for 2, 4, 6, and 18 h before the total RNA was extracted. PKCδ inhibition had no effect on iNOS mRNA decay in quantitative RT-PCR analysis (Fig. 4C).

### Effects of PKCδ on transcription factor NF-κB

**Figure 5:**
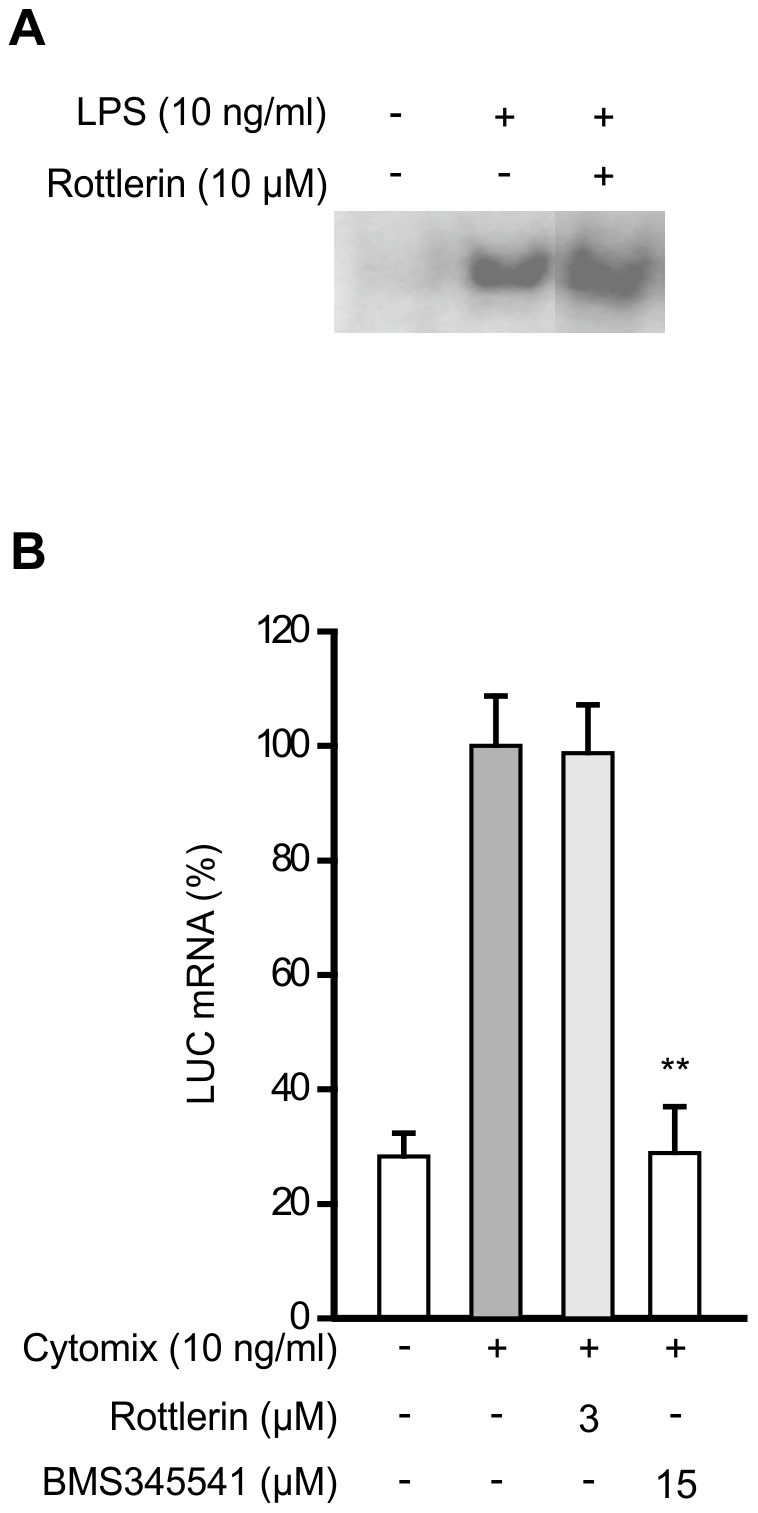
Effect of PKCδ on NF-κB activity. (A) J774 cells were stimulated with LPS and treated with rottlerin for 30 min before the preparation of nuclear extracts. NF-κB DNA binding activity was analyzed by EMSA. Gels shown are representatives of three others with similar results. (B) L929 cells were transfected with NF-κB reporter to form L929 pNF-κB cell line. The transfected cells were stimulated with a combination of cytokines (IL-1β, IFNγ, and TNFα) and treated with rottlerin for 1 h before the total mRNA was extracted and subjected to RT-PCR. Luciferase mRNA levels were normalized against GAPDH mRNA. IκB kinase inhibitor BMS3445541 was used as a control agent to inhibit NF-κB mediated transcription. Values are mean+SEM, n = 4. **p<0.01.

To evaluate whether the effect of PKCδ inhibition on iNOS mRNA expression could be due to its effects on nuclear factor κB, which is an important transcription factor for iNOS [8], we measured the effect of PKCδ inhibition on the activation of NF-κB by EMSA. Rottlerin had no effect on LPS-induced NF-κB activation or binding activity (Fig. 5A).

In addition, the effect of rottlerin was studied on NF-κB mediated transcription in L929 pNFκB cell line, which was stably transfected with luciferase reporter gene under the control of NF-κB-responsive promoter. Similarly to results obtained from EMSA, inhibition of PKCδ by rottlerin had no effect on NF-κB-dependent transcription induced by the mixture of cytokines, while IκB kinase inhibitor BMS3445541 inhibited luciferase mRNA expression (Fig. 5B).

### Effects of PKCδ on transcription factors IRF1 and STAT1

**Figure 6:**
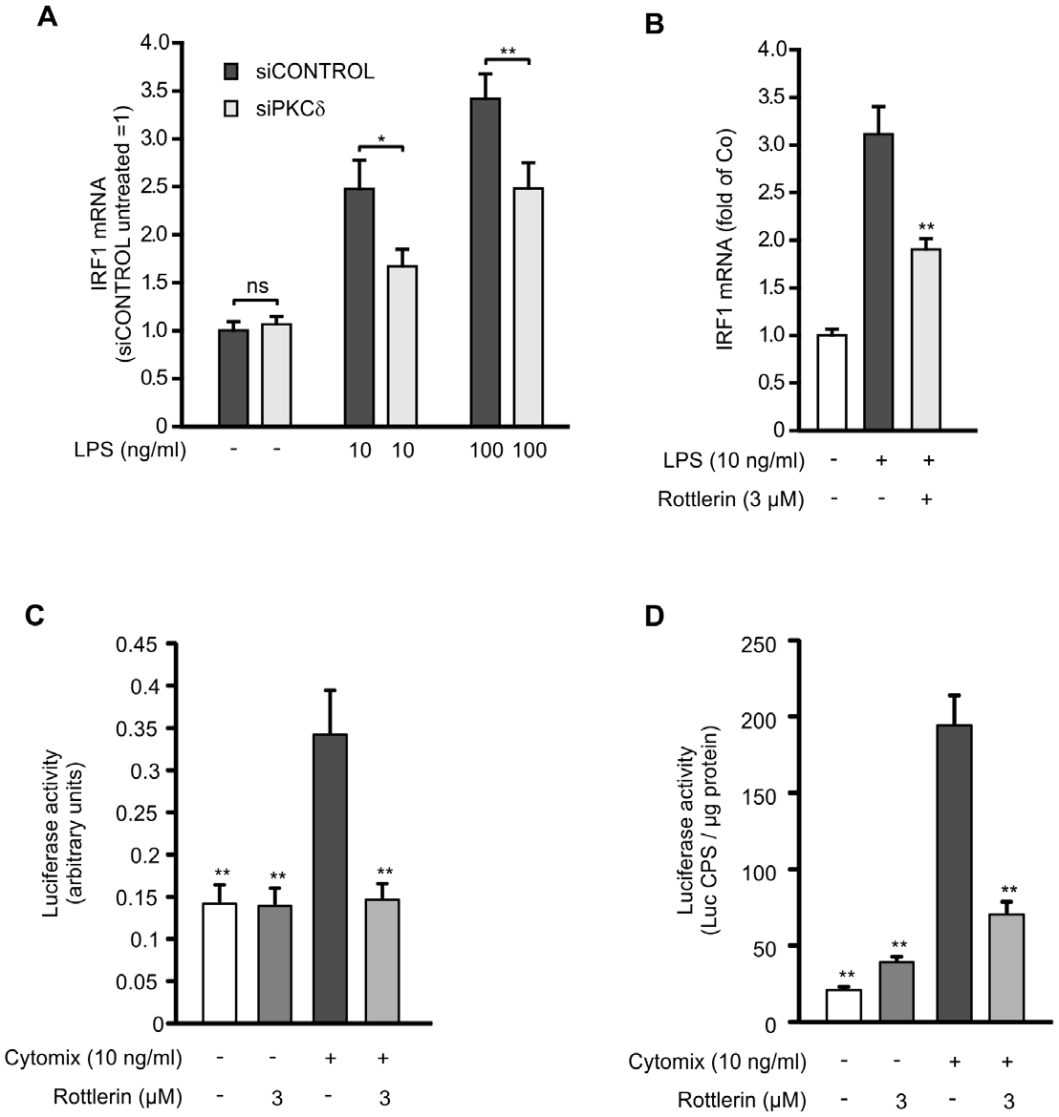
Effect of PKCδ on the expression of transcription factor IRF1 and on IRF1 and STAT1 mediated transcription. (A) J774 macrophages were transiently transfected with PKCδ siRNA using DharmaFECT 4 transfection reagent and treatment with non-targeting siRNA was used as control. Macrophages were stimulated with LPS for 4 h before incubations were terminated and extracted total RNA was subjected to RT-PCR. IRF1 mRNA levels were normalized against GAPDH mRNA. Values are mean+SEM, n = 6. (B) J774 macrophages were stimulated with LPS and treated with rottlerin for 4 h before incubations were terminated and extracted total RNA was subjected to RT-PCR. IRF1 mRNA levels were normalized against GAPDH mRNA. Values are mean+SEM, n = 3. (C) L929 cells were transfected with IRF1 reporter. At 24 h post transfection culture media was changed and cells were incubated with compounds of interest for 6 h. Firefly and *Renilla* luciferase activities were then measured by luminometer using Dual-Glo®Luciferase Assay System. Firefly luciferase activity was normalized to *Renilla* luciferase activity. Values are mean+SEM, n = 8–10. (D) L929 cells were transiently transfected with STAT1 reporter. At 24 h post transfection culture media was changed and cells were incubated with compounds of interest for 6 h. Thereafter luciferase activity in cell lysates was measured by luminometer using Luciferase Assay System. Values are mean+SEM, n = 4. *p<0.05, **p<0.01.

IRF1 is an important transcription factor for iNOS, but in contrast to NF-κB, IRF1 has been shown to act as a later phase transcription factor [7], [9]. Since the pharmacological inhibition or siRNA-mediated down-regulation of PKCδ decreased iNOS mRNA levels particularly in the later time points, we studied the effects of PKCδ on IRF1. Silencing of PKCδ by siRNA as well as PKCδ inhibitor rottlerin suppressed IRF1 expression in cells activated with LPS (Fig. 6A–B). To study if the inhibition of IRF1 expression would have expected functional consequences, we measured the effect of PKCδ inhibitor rottlerin on the transcriptional activity of IRF1 in L929 cells transfected with constructs containing luciferase reporter gene under the control of IRF1-responsive promoter. Rottlerin clearly inhibited cytokine-induced IRF1-dependent transcription in the reporter gene experiments (Fig. 6C).

In cells exposed to inflammatory stimuli, IRF1 has been shown to be up-regulated partly directly and partly through enhanced STAT1 activation [31]. Therefore we investigated the effects of PKCδ inhibitor rottlerin on STAT1 mediated transcription in L929 cells. Cells were transiently transfected with a construct containing luciferase reporter gene under the control of STAT1-responsive promoter. STAT1-mediated transcription was significantly increased when cytokines were added into the culture and PKCδ inhibitor rottlerin inhibited the CM-induced STAT1-dependent transcription when measured by the reporter gene assay in L929 cells (Fig. 6D).

### Effects of IRF1 siRNA on the expression of iNOS, IL-6 and TNFα

**Figure 7:**
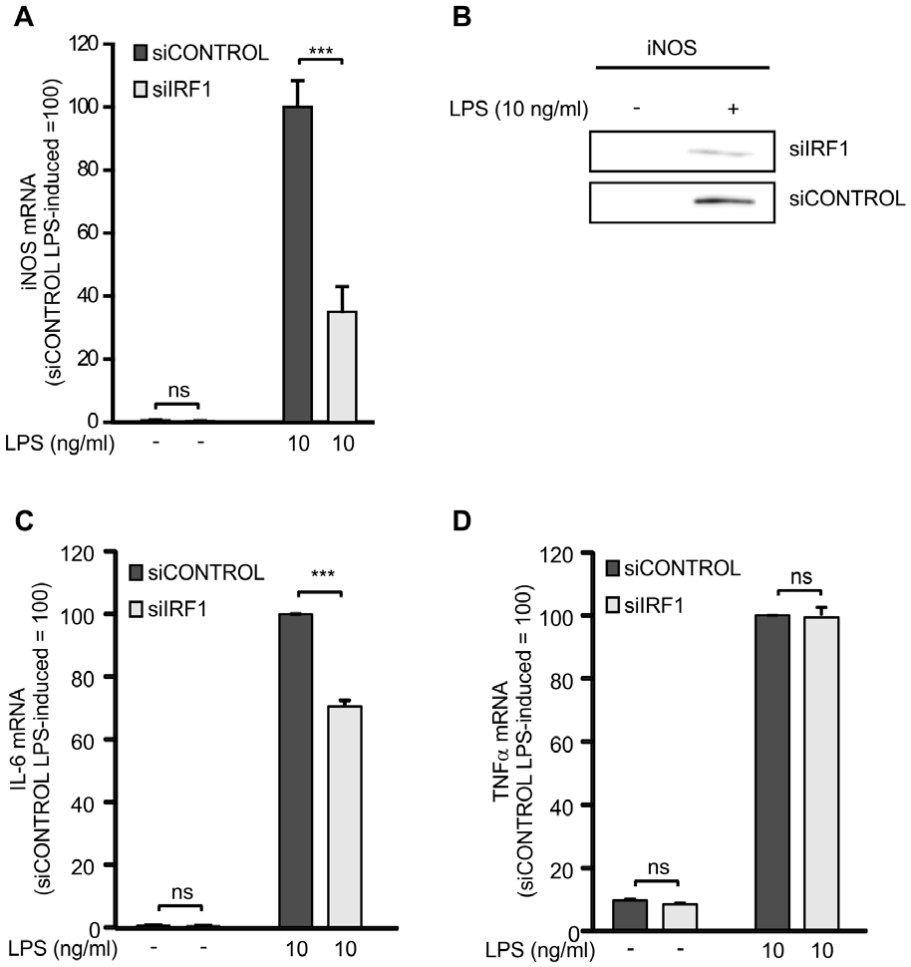
Effect of IRF1 on the expression of iNOS, IL-6 and TNFα. (A) J774 macrophages were transiently transfected with IRF1 siRNA using DharmaFECT 4 transfection reagent and treatment with non-targeting siRNA was used as control. Macrophages were stimulated with LPS for 4 h before incubations were terminated and extracted total RNA was subjected to RT-PCR to measure iNOS mRNA expression. iNOS mRNA levels were normalized against GAPDH mRNA. Values are mean+SEM, n = 4–5. (B) J774 macrophages were transiently transfected with IRF1 siRNA using DharmaFECT 4 transfection reagent and treatment with non-targeting siRNA was used as control. Thereafter the macrophages were stimulated with LPS. After 24 h, incubations were terminated and immunoblots were run using iNOS specific antibody. Gels shown are representatives of three others with similar results. (C–D) J774 macrophages were transiently transfected with IRF1 siRNA using DharmaFECT 4 transfection reagent and treatment with non-targeting siRNA was used as control. Macrophages were stimulated with LPS for 8 h (C) and 3 h (D) before incubations were terminated and extracted total RNA was subjected to RT-PCR to measure IL-6 (C) and TNFα (D) mRNA expression. IL-6 and TNFα mRNA levels were normalized against GAPDH mRNA. Values are mean+SEM, n = 4. ***p<0.001.

To confirm the role of transcription factor IRF1 in iNOS expression, we studied the effects of down-regulation of IRF1 by siRNA on iNOS mRNA and protein expression. IRF1 siRNA resulted in more than 80% suppression in IRF1 protein levels in LPS treated cells (Figure S1). Down-regulation of IRF1 by siRNA clearly inhibited LPS-induced iNOS mRNA (Fig. 7A) and protein expression (Fig. 7B). We investigated the effects of IRF1 down-regulation also on IL-6 and TNFα expression because IRF1 has been shown to be an important transcription factor for IL-6 but not to regulate TNFα expression [7], [32]. Indeed, down-regulation of IRF1 by siRNA inhibited also LPS-induced IL-6 but not TNFα mRNA expression (Fig. 7C–D).

### Effects of PKCδ on IL-6 and TNFα production

**Figure 8:**
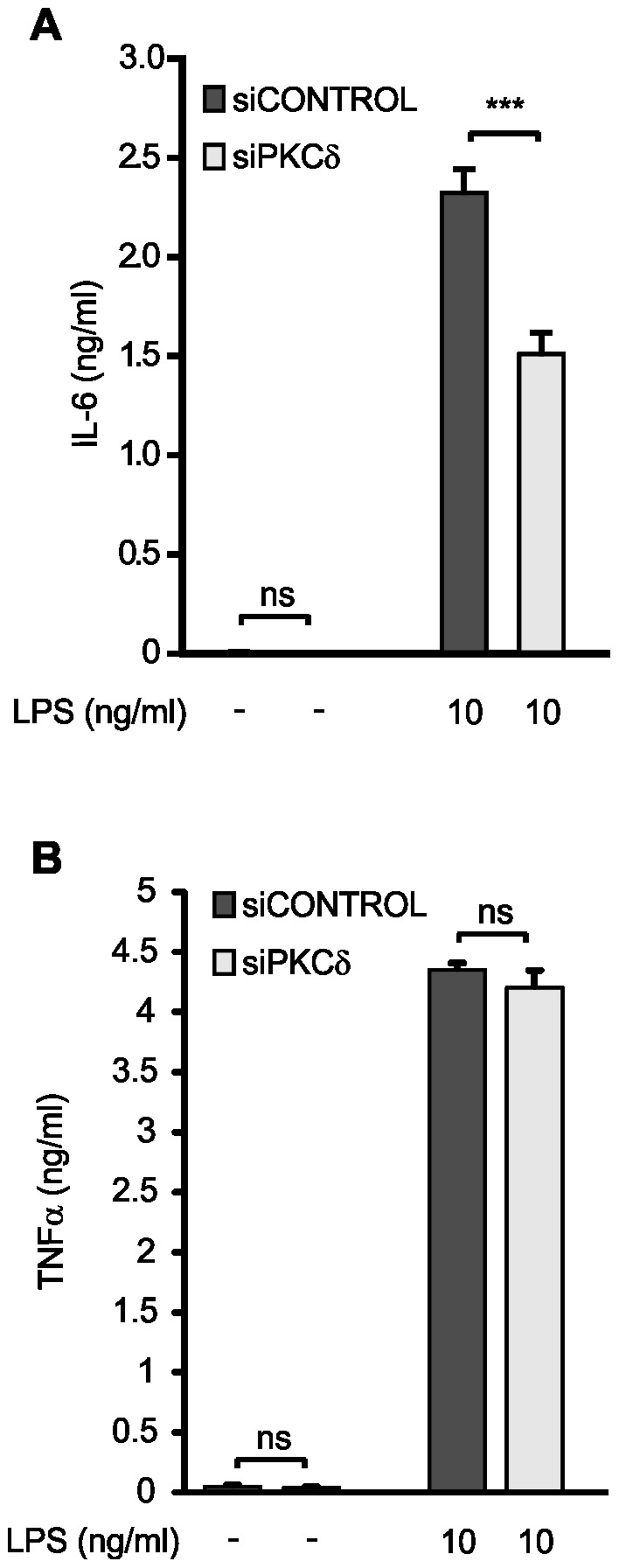
Effect of PKCδ on IL-6 and TNFα production. J774 macrophages were transiently transfected with PKCδ siRNA using DharmaFECT 4 transfection reagent. Treatment with non-targeting siRNA was used as control. Macrophages were stimulated with LPS for 24 h before incubations were terminated and IL-6 (A) and TNFα production (B) was determined by ELISA. Values are mean+SEM, n = 6 (A), n = 3 (B), ***p<0.001.

In order to study whether PKCδ affects the production of other IRF1-responsive inflammatory factors, we measured the production of IL-6 in cells treated with PKCδ siRNA. Down-regulation of PKCδ by siRNA decreased LPS-induced IL-6 production when compared to cells treated with non-targeting control siRNA (Fig. 8A). In contrast, down-regulation of PKCδ by siRNA had no effect on LPS-induced TNFα production (Fig. 8B) mimicking the effects of IRF1 down-regulation.

### Effects of rottlerin on carrageenan induced paw inflammation in mice

**Figure 9:**
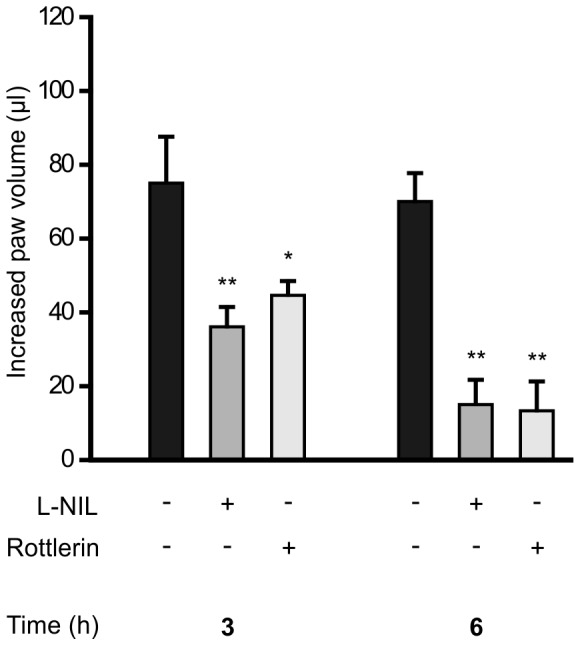
Effect of rottlerin on carrageenan induced paw inflammation model in mice. L-NIL and rottlerin were administered i.p. 2 h prior to carrageenan (1.5%) was injected into a hind paw. Paw edema was measured before, 3 h and 6 h after carrageenan injection. Edema is expressed as the difference between the volume changes of the carrageenan treated paw and the control paw (injected with saline). Values are mean+SEM, n = 5–6, **p<0.01.

In order to investigate if the anti-inflammatory effects of inhibition of PKCδ could be translated also to an *in vivo* situation, we investigated the effects of rottlerin in comparison to iNOS inhibitor L-NIL in carrageenan-induced paw inflammation in the mouse. Intradermal injection of carrageenan has been reported to cause an acute inflammatory response which is partly mediated by increased NO production as evidenced by the anti-inflammatory effect of iNOS inhibitors in the model [33], [34]. That was also true in our hands. Intraperitoneal administration of iNOS inhibitor L-NIL (50 mg/kg) reduced carrageenan induced paw edema by 50% and 75% when measured three and six hours following carrageenan injection, respectively. Interestingly, PKCδ inhibitor rottlerin (10 mg/ml) had also a very clear anti-inflammatory effect, which resembled that of L-NIL and achieved over 75% inhibition of the carrageenan induced paw inflammation when measured at six hours following carrageenan injection (Fig. 9).

## Discussion and Conclusions

PKCδ is known to regulate lymphocyte functions, but less is known about its effects in innate immunity and on the regulation of the expression of inflammatory genes. In the present study, we investigated the role of a novel isoenzyme PKCδ in the regulation of iNOS expression and NO production in inflammatory conditions by using PKCδ targeted siRNA and a pharmacological PKCδ inhibitor. The results suggest that PKCδ contributes to the induction of IRF1, possible through activation of STAT1, and by this way enhanced the expression of iNOS. We also show that the inhibition of PKCδ attenuated the acute inflammatory response in carrageenan induced paw inflammation *in vivo* as did the iNOS inhibitor L-NIL.

Stimulation of J774 macrophages with LPS induced iNOS expression and NO production and they were reduced by silencing PKCδ with siRNA and by inhibiting of PKCδ by rottlerin. In cells where PKCδ had been down-regulated by siRNA, rottlerin had no effect on iNOS expression. Rottlerin and PKCδ siRNA had similar effects also in L929 fibroblasts, where they were shown to reduce cytokine-induced NO production and iNOS expression. Rottlerin, a natural compound isolated from *Mallotus phillippinensis*, was first described as a PKCδ selective inhibitor by Gschwendt and coworkers [35]. Later, it has been reported that rottlerin inhibits also other kinases, e.g. PDK1 and PKA [36], [37]. In the current study, the inhibitory effect of rottlerin on iNOS expression and NO production seemed to be mediated mostly through PKCδ, since the effects of rottlerin were in line with the effects of PKCδ specific siRNA in two different cell lines. Furthermore, rottlerin did not have additional inhibitory effect on iNOS expression in cells in which PKCδ had been down-regulated with siRNA.

In the further experiments, the effect of PKCδ silencing on the expression of iNOS mRNA was evaluated. In these experiments, rottlerin and PKCδ siRNA had no effect on iNOS mRNA expression when measured at the early time points, but reduced iNOS mRNA expression at the later time points. It is known that iNOS expression is regulated by post-transcriptional mechanisms, especially through the regulation of iNOS mRNA stability. Indeed, the 3′-untranslated region of murine iNOS mRNA contains adenylate- and uridylate- (AU-) rich elements, which are known to control mRNA stability of many transiently expressed genes [38]–[40]. We have previously shown that glucocorticoid dexamethasone [27] and the inhibition of JNK signaling [29], [41] reduced iNOS expression by enhancing iNOS mRNA decay. However, in the present study PKCδ inhibitor rottlerin was not found to affect iNOS mRNA decay, suggesting that PKCδ does not regulate iNOS mRNA stability in the inflammatory conditions studied.

Regulation of protein degradation is another mechanism of post-transcriptional regulation of iNOS expression. Since iNOS protein has been shown to be degraded by the proteasome [24]–[26], we studied whether the effect of rottlerin could be reversed with proteasome inhibitor lactacystin [42]. Lactacystin itself enhanced iNOS protein levels, as expected and as previously reported [24]–[26]. Lactacystin, however, did not reverse or diminish the effect of rottlerin. This suggested that the reduction of iNOS expression by rottlerin is not due to enhanced proteasomal degradation of iNOS protein.

The expression of iNOS is tightly regulated also at the transcriptional level. One of the most important transcription factors for iNOS, especially at the early phase of transcription, is NF-κB [8], [43]. In our hands, rottlerin had no effect on the activation of NF-κB or on NF-κB mediated transcription, although PKCδ has previously been shown to regulate iNOS expression in an NF-κB dependent manner [44]. IRF1 is another key transcription factor for iNOS. In macrophages and glial cells from IRF1 knockout mice, LPS and IFNγ-induced iNOS mRNA expression was markedly reduced when compared to wild type cells [7], [9]. However, the role of IRF1 in iNOS induction seems to be tissue-specific, since IRF1 is not required for iNOS expression in murine chondrocytes or pancreatic islet cells [45], [46]. Unlike NF-κB, IRF1 is not constitutively present in the cytosol in an inactive form, but it is synthesized *de novo* by inflammatory stimulation [47]. Thus, the activation of IRF1 is usually slower than that of NF-κB. In J774 macrophages, a marked increase in IRF1 mRNA was seen 4 h after addition of LPS, so IRF1 can well be considered as a later phase transcription factor for iNOS. Interestingly, IRF1 expression was clearly reduced by both the down-regulation of PKCδ by siRNA and by the PKCδ inhibitor rottlerin. The signaling pathway leading to activation of IRF1 has been studied with the emphasis on IFN stimulated cells [48], [49]. It has been shown, by using macrophages from STAT1 deficient mice, that INFγ, and to a lesser extent IFN
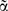
β, induce IRF1 gene expression through activation of STAT1 [49]–[51]. Gao et al. [31] proposed that when murine macrophages were stimulated with LPS, IRF1 was activated partly directly and partly through increased synthesis of IFNάβ and activation of STAT1. Therefore, we studied the effects of PKCδ inhibitor rottlerin on STAT1 activation. We observed a reduction in STAT1 mediated transcription as measured by STAT1 reporter assay. These results suggest that the effects of PKCδ on iNOS expression could be mediated through activation of STAT1 leading to the enhanced expression and activity of IRF1, and finally, induction of iNOS.

IRF1 regulates also the production of IL-6 [32]. In our studies, the down-regulation of IRF1 or PKCδ by siRNA inhibited IL-6 expression. On the other hand, the production of TNFα has been shown not to be regulated by IRF1 [7]. Accordingly, TNFα expression in the present study was not affected in cells treated with IRF1 siRNA or PKCδ siRNA. These results further support the conclusion that PKCδ regulates the expression of transcription factor IRF1 and by this way contributes to the regulation of the expression of IRF1-responsive inflammatory factors including iNOS.

Further, we investigated whether the anti-inflammatory effects of rottlerin seen *in vitro* could be translated to *in vivo* conditions. We investigated the effects of rottlerin on carrageenan induced acute paw inflammation model in the mouse. Intradermal injection of carrageenan causes an acute inflammatory response, and this response has been reported to be partly mediated by increased NO production as shown by the anti-inflammatory effect of iNOS inhibitors [33], [34]. In the present study, iNOS inhibitor L-NIL reduced the carrageenan induced acute inflammatory edema, confirming that NO is involved in this inflammatory response. Rottlerin showed to be effective also *in vivo* and it reduced the carrageenan induced acute inflammatory paw inflammation mimicking the effect of iNOS inhibitor L-NIL. In animal studies using iNOS inhibitors or iNOS deficient mice, iNOS-dependent NO production has been reported to contribute also to many other inflammatory conditions, including lung inflammation and the development of acute lung injury [52]–[54]. Also, lungs of the patients with acute respiratory distress syndrome display high levels of iNOS as well as extensive nitrotyrosine staining, which is a marker of iNOS-dependent NO production in tissues [55]. Interestingly, the inhibition of PKCδ has also been shown to suppress acute lung inflammation. Intratracheal administration of a specific PKCδ-TAT peptide inhibitor lowered inflammatory cytokine and chemokine levels in plasma and attenuated the infiltration of inflammatory cells to the lung tissue and the disruption of lung tissue architecture due to polymicrobial sepsis in mice [56]. Our results support the assumption that PKCδ participates in the regulation of acute inflammatory response in addition to adaptive immunity. Further, it is also possible that PKCδ inhibitors limit the development of tissue injury in acute inflammation by attenuation of iNOS expression and NO production. In the present study, we also found that PKCδ regulates the expression of IRF1 and IRF1 was found to mediate the effects of PKCδ on iNOS expression. IRF1 is also a key factor in the regulation of adaptive immunity [57]. Whether PKCδ participates in the regulation of adaptive immune response through IRF1, needs further studies.

In conclusions, we have shown that PKCδ participates in the regulation of NO production and iNOS expression in activated macrophages and fibroblasts. We showed, for the first time, that PKCδ up-regulates transcription factor IRF1, possibly through activation of transcription factor STAT1. IRF1 then enhances the expression of iNOS, and most likely also other IRF1-dependent genes, such as IL-6. In addition, inhibition of PKCδ was found to have anti-inflammatory properties also *in vivo*. Taken together, these results suggest that PKCδ inhibitors hold anti-inflammatory properties *in vitro* and *in vivo*, making PKCδ a potential target for anti-inflammatory drug development.

## Supporting Information

Figure S1 **Downregulation of PKCδ and IRF1 by siRNA.** J774 macrophages (A) and L929 fibroblasts (B) were transiently transfected with PKCδ specific siRNA. (C) J774 macrophages were transiently transfected with IRF1 specific siRNA. Non-targeting siRNA (siCONTROL) was used as a control. The gels shown are representatives of three others with similar results.
(TIF)

Table S1 **Primer and probe sequences.**
(DOCX)
