# Supplementary figures and images for: Down-Regulation of Protein Kinase Cδ Inhibits Inducible Nitric Oxide Synthase Expression through IRF1
Source: PLoS One. 2013 Jan 9;8(1):e52741. doi: 10.1371/journal.pone.0052741 (PMC3541401; doi:10.1371/journal.pone.0052741)

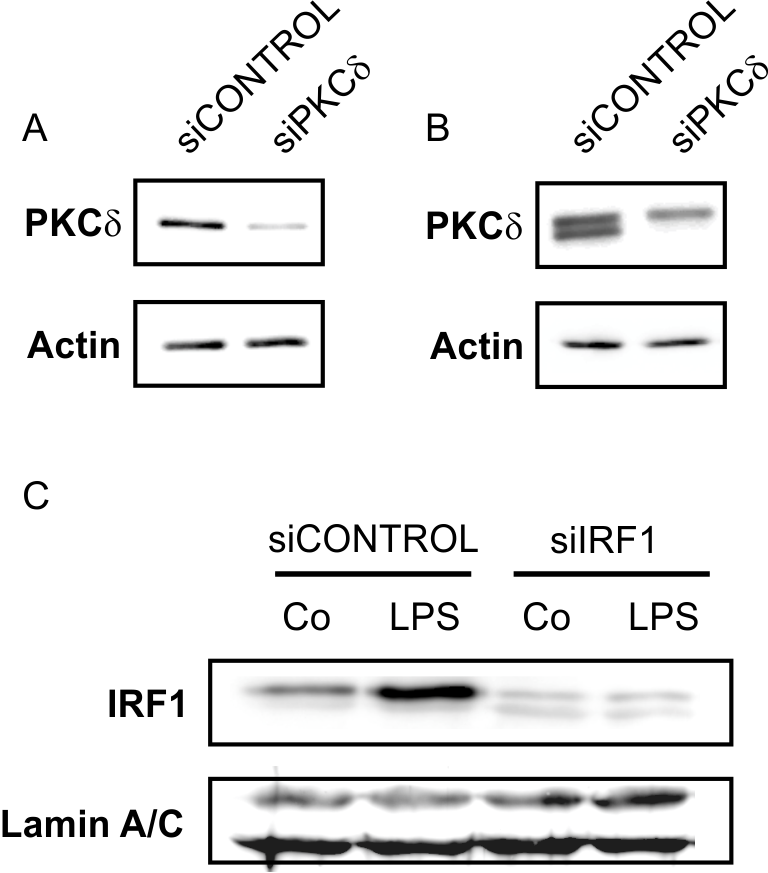

Supplement: Figure S1 — Downregulation of PKCδ and IRF1 by siRNA. J774 macrophages (A) and L929 fibroblasts (B) were transiently transfected with PKCδ specific siRNA. (C) J774 macrophages were transiently transfected with IRF1 specific siRNA. Non-targeting siRNA (siCONTROL) was used as a control. The gels shown are representatives of three others with similar results. (TIF) [file pone.0052741.s001.tif]
