# Supplementary material for: Down-Regulation of Protein Kinase Cδ Inhibits Inducible Nitric Oxide Synthase Expression through IRF1
Source: PLoS One. 2013 Jan 9;8(1):e52741. doi: 10.1371/journal.pone.0052741 (PMC3541401; doi:10.1371/journal.pone.0052741)
Supplement: Table S1 — Primer and probe sequences. (DOCX) [file pone.0052741.s002.docx]

| **Gene** | **Oligonucleotide** | **Sequence 5’ → 3’** |
| --- | --- | --- |
| Murine iNOS | Forward primer | CCTGGTACGGGCATTGCT |
|  | Reverse primer | GCTCATGCGGCCTCCTT |
|  | Probe | CAGCAGCGGCTCCATGACTCCC |
| Murine GAPDH | Forward primer | GCATGGCCTTCCGTGTTC |
|  | Reverse primer | GATGTCATCATACTTGGCAGGTTT |
|  | Probe | TCGTGGATCTGACGTGCCGCC |
| Murine IL-6 | Forward primer | TCGGAGGCTTAATTACACATGTTC |
|  | Reverse primer | CAAGTGCATCATCGTTGTTCATAC |
|  | Probe | CAGAATTGCCATTGCACAACTCTTTTCTCA |
| Murine TNFα | Forward primer | AATGGCCTCCCTCTCATCAGTT |
|  | Reverse primer | TCCTCCACTTGGTGGTTTGC |
|  | Probe | CTCAAAATTCGAGTGACAAGCCTGTAGCCC |
| Luciferase | Forward primer | AAAAAGTTGCGCGGAGGAG |
|  | Reverse primer | TTTTTCTTGCGTCGAGTTTTCC |
|  | Probe | TGTGTTTGTGGACGAAGTACCGAAAGGTCTTAC |
